# Ultra-broadband spectrometer on a chip of picometer scale resolution

**DOI:** 10.1038/s41377-023-01280-6

**Published:** 2023-09-15

**Authors:** Alina Karabchevsky

**Affiliations:** School of Electrical and Computer Engineering, Ben-Gurion University of the Negev, Beer-Sheva, 8410501 Israel

**Keywords:** Optical spectroscopy, Integrated optics

## Abstract

A reconfigurable photonic integrated circuit was developed to operate as an ultra-broadband spectrometer on SiN chip resolving spectral lines with picometer precision and thermal stability of ±2 °C.

## Abstract (web summary)

Since its creation, the reduction in size of the spectroscope has resulted in a balance between resolution, bandwidth, and signal-to-noise ratio. Up until now, downsized demonstrations have struggled to surmount the technical hurdles of achieving both extremely high resolution (reaching the scale of picometers) and wide bandwidth (exceeding 100 nanometers) at the same time. Nonetheless, these capabilities are fundamental prerequisites for analytical spectroscopy tools in diverse applications such as biomedical sensing and industrial chemical monitoring. They are also critical for compact optical imaging systems like spectral-domain optical coherence tomography (SD-OCT), which demand substantial imaging depth and superior spatial resolution.

## Graphical abstract

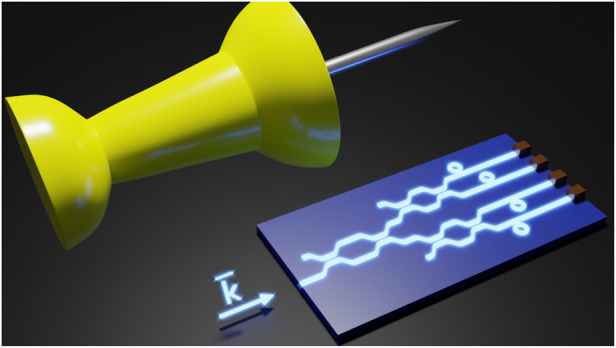

Optical spectroscopy has played a crucial role in various historical discoveries, such as identifying ancient artifacts with carvings resembling friction ridge skin found in numerous locations worldwide. Additionally, prehistoric picture writing featuring handprints with ridge patterns was discovered on a cliff in Nova Scotia and other regions, thanks to the insights provided by optical spectroscopy. Even in ancient Babylon, fingerprints were utilized on clay tablets for business transactions, showcasing the long-standing significance of this spectroscopic technique in uncovering and understanding ancient practices and artifacts.

The optical spectroscope was invented in 1859 by Robert Bunsen and Gustav Kirchhoff^1^. Their pioneering work in 1860 attributed fingerprints as chemical elements. Kirchhoff, in particular, suggested that by observing the emission spectra of flames through a dispersive element like a prism, it might be possible to differentiate flames with similar colors. When he passed bright light through these flames, the dark lines in the absorption spectrum of the light corresponded to the wavelengths of the bright, sharp lines characteristic of the emission spectra of the same test materials. Despite facing an unknown disability that confined him to a wheelchair or crutches for most of his life, Kirchhoff conducted groundbreaking research that significantly contributed to the fundamental understanding of electrical circuits and the development of optical spectroscopy. The last stands as one of the most powerful and widely used characterization tools^2–6^.

Since the inception of the spectroscope, its size reduction has led to trade-offs between resolution, bandwidth, and signal-to-noise ratio. Up to now, scaled-down demonstrations have not been able to overcome the technical challenges of achieving both ultra-high resolution (down to picometer-scale) and broad bandwidth (>100 nm) simultaneously^6,7^. Nevertheless, these capabilities are essential requirements for analytical spectroscopy tools in various biomedical sensing^8^ and industrial chemical monitoring applications^9^, as well as for miniaturized optical imaging systems like spectral-domain optical coherence tomography (SD-OCT) that necessitate large imaging depth and high spatial resolution^10^. Efforts have been made to develop miniaturized resonant spectrometers (RSs) based on passive spectrum filters, utilizing disordered scattering media, metasurfaces, photonic crystals, or quantum dot-based filter arrays^11–13^, metasurface-, photonic crystal-, or quantum dot-based filter arrays^14^. While these represent a compact form of RS, the number of channels can be limited due to passive splitting loss. Recently, active RSs with tunable spectral responses have been explored, including detector-only RSs with tunable absorption spectra and filter-based RSs using MEMS or thermally tunable resonators^15,16^. However, the methods of generating sampling channels using lumped structures have demonstrated limited decorrelation, posing challenges to achieve an ultra-high bandwidth-to-resolution ratio as per the principles of compressive sensing^17^.

In their paper, Qixiang Cheng, Richard Penty^18^, and co-authors present a noteworthy advancement towards achieving an ultra-broadband picometer-scale resolution spectrometer on a photonic integrated chip. They introduced a novel method that utilizes distributed filters to generate ultra-broadband pseudo-random spectral responses. To ensure thermal robustness, the photonic integrated chip is fabricated using a CMOS-compatible silicon nitride (SiN) platform, as temperature variations can limit the reconstruction accuracy when aiming for picometer-scale resolution. This work represents a significant step forward in developing highly capable and compact spectrometers for a wide range of applications (Fig. 1).

**Fig. 1:**
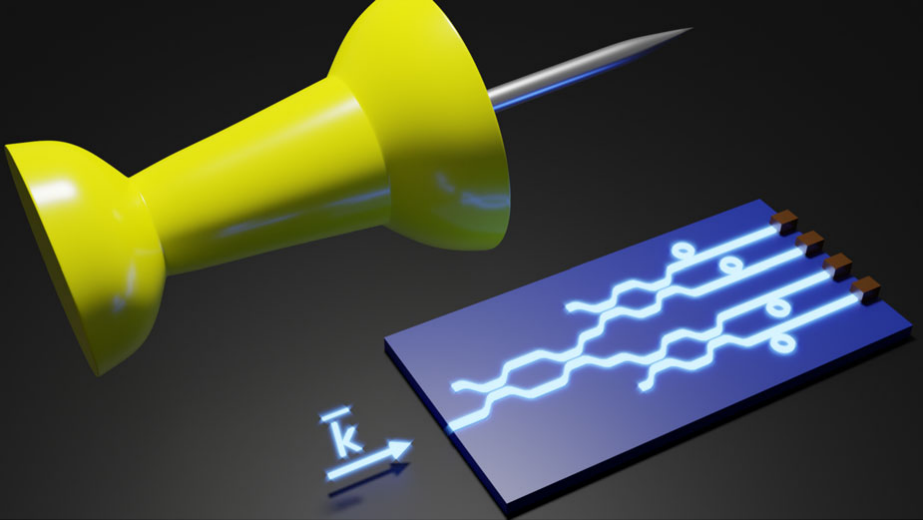
The schematic view illustrates an ultra-tiny spectrometer designed to fit on a microchip. For scale comparison, the device is shown alongside a thumbtack. The direction of the light is indicated by the vector *k*

As a result of the introduced distributed filters in a reconfigurable photonic network on a chip, the sampling channels become highly uncorrelated, facilitating computational reconstruction. The utilization of distributed filters in the photonic network offers excellent scalability in the number of sampling channels without compromising their decorrelation. By carefully engineering the spectral properties of each distributed filter, the overlaid transmission spectra can form a sampling matrix with minimal self- and cross-correlation. This design enables effective information acquisition across the entire spectrum. Moreover, embedding filters in the reconfigurable photonic network allows for spectrum shaping, enhancing the versatility and performance of the spectrometer.

Reconfigurable photonics allows for user-defined performance, providing an additional level of programmability that depends on the trade-offs between resolution, computation complexity, and relative error. This enhanced programmability broadens the applications of the technology, catering to various use cases. It can be employed for identifying signature spectral peaks with acceptable levels of performance, as well as for relative metrology demanding ultra-high resolution and minimal errors^19^. This flexibility in performance customization makes reconfigurable photonics a versatile solution with widespread potential in different fields and applications.
